# Proposal for an index to evaluate dichotomous keys

**DOI:** 10.3897/zookeys.685.13625

**Published:** 2017-07-17

**Authors:** Nguyen Van Sinh, Martin Wiemers, Josef Settele

**Affiliations:** 1 Institute of Ecology and Biological Resources (IEBR), Vietnam Academy of Science and Technology (VAST), 18 Hoang Quoc Viet, Nghia Do, Cau Giay, Ha Noi, Vietnam; 2 Graduate School of Science and Technology, Vietnam Academy of Science and Technology (VAST), 18 Hoang Quoc Viet, Nghia Do, Cau Giay, Ha Noi, Vietnam; 3 Helmholtz Centre for Environmental Research – UFZ, Dept. Community Ecology, Theodor-Lieser-Str. 4, 06120 Halle, Germany; 4 Institute of Biological Sciences, University of the Philippines Los Baños, College of Arts and Sciences, Laguna 4031, Philippines

**Keywords:** index, dichotomous key, evaluation

## Abstract

Dichotomous keys are the most popular type of identification keys. Studies have been conducted to evaluate dichotomous keys in many aspects. In this paper we propose an index for quantitative evaluation of dichotomous keys (E_dicho_). The index is based on the evenness and allows comparing identification keys of different sizes.

## Introduction

**Figure 1.**
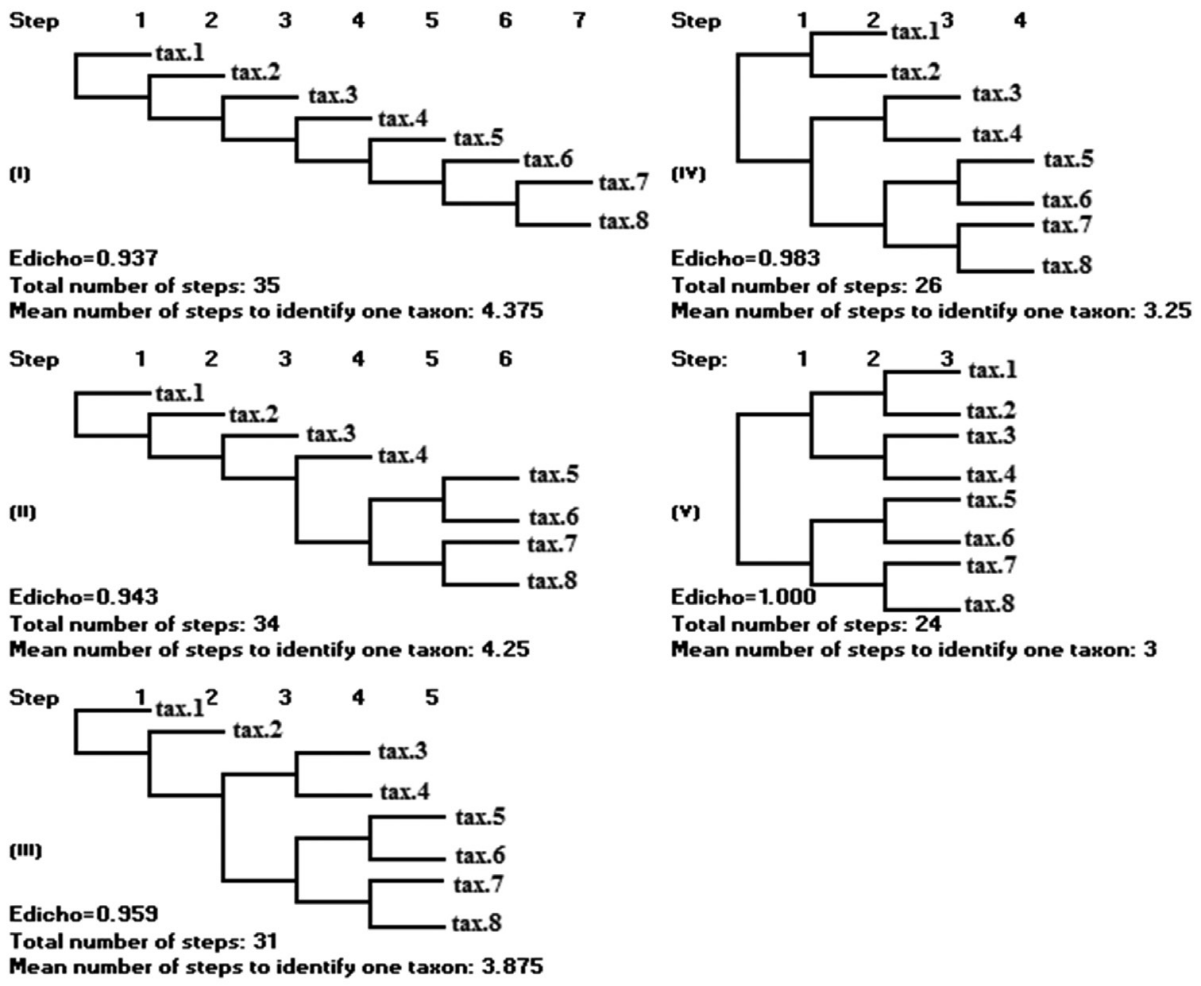
Schematic presentation of 5 dichotomous keys for a group of 8 taxa.

A taxonomic key is a method used to identify organisms. Dichotomous keys are the most popular type of identification keys. Dichotomous keys are single entry identification keys. They consist of nested questions or couplets, and each question provides two choices or leads (Thesis and Antithesis). These choices contain descriptions of key characteristics of an organism. The paired statements or choices consider the differences between items. After choosing the statement that best matches the object, the user proceeds to another pair of statements until the name of the taxon is identified. There may be several keys for a group of taxa. This prompts the question, which key has a better performance, provided that all the used characters are good ones which allow an unambiguous identification? How can we evaluate quantitatively the performance of the keys? As a key is intended for identification of each of the taxa in the group, the key will achieve the highest performance when the mean number of steps to their identification is minimal. If the number of steps to identification of the taxa in a key become more even, the mean number of steps to their identification is decreasing, and the mean number of steps to identification of the taxa is minimal when the number of steps to their identification are most even (Fig. 1). These considerations lead us to the evenness index of Pielou (1966). This paper proposes an index that is based on Pielou’s evenness index for quantitative evaluation of dichotomous keys.

## Methods

We use Pielou’s evenness index as a prototype for our index. Pielou’s evenness index (J) can be calculated using the following formula (Help et al. 1998):

**Figure:**
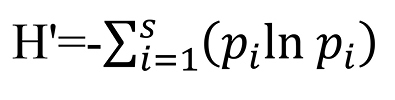


where:

- H’ is the Shannon diversity index. This measure was originally proposed by Shannon (1948) to quantify the entropy (uncertainty or information content) in strings of text. The idea is that the more different the letters are, and the more equal their proportional abundances in the string of interest, the more difficult it is to correctly predict which letter will be the next one in the string. The index can be calculated using the following formula:

**Figure:**
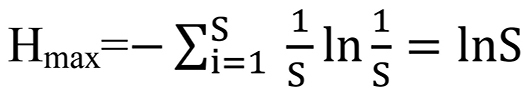


In which *p_i_* is the proportion of characters belonging to the *i*th type of letter in the string of interest and *S* the number of types of letter.

- H_max_ is the maximum value of H’ and equal to:

**Figure:**
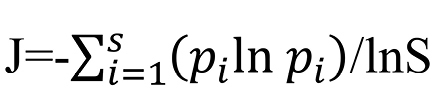


As result, Pielou’s evenness index can be calculated according to the following formula:

**Figure:**
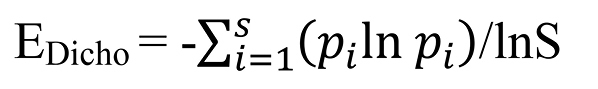


## Results and discussion

If the number of steps we have to pass to come to a decision (a taxon) is N_i_ and the total steps when we identify all the taxa is N, the proportion of the steps to identify the *i*th taxon is equal:

*p_i_* = N_i_/N

**Figure 2.**
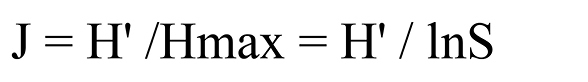
Schematic presentation of a dichotomous key.

As can be inferred from the scheme of a dichotomous key (Fig. 2), the number of taxa in a dichotomous key corresponds to S − the number of types of letters in the formula of Pielou’s evenness index.

We call the index for dichotomous keys E_Dicho_ (because of its origin from evenness index). As a result, E_Dicho_ is equal:

**Figure:**
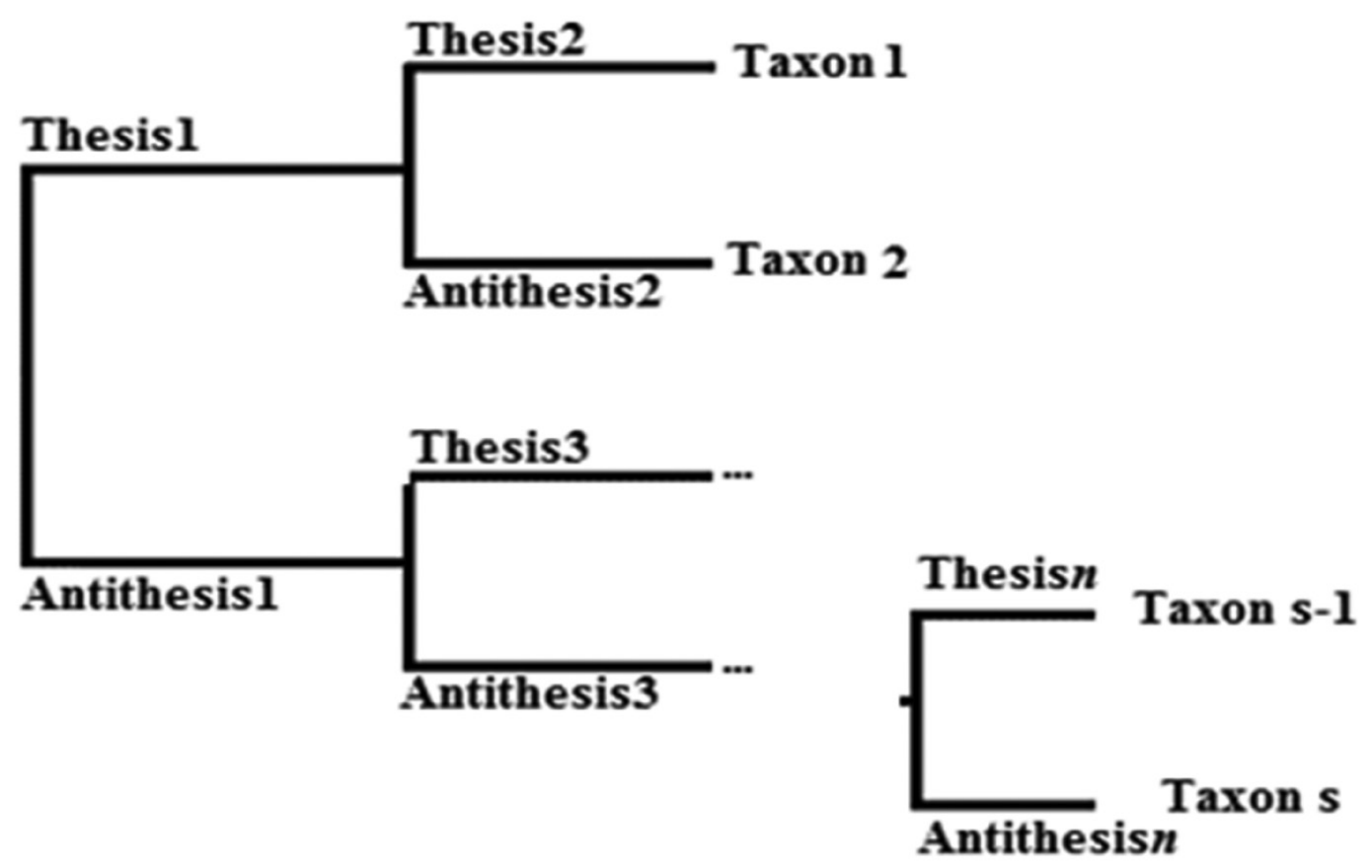


Where: S is the number of taxa of the key, and *p_i_* is the proportion of steps to identify the *i*th taxon.

Many attempts have been undertaken in order to evaluate identification keys (e.g. Lobanov 1975, 1983, 2015, Pankhurst 1978, Leuschner and Sviridov 1986, Leuschner 1991). Generally, these are methods that are based on the same concept of average length of taxon definition in a key and comparison of this number with the theoretical minimum. However, these attempts do not consider the length evenness of taxon definitions.

Several studies have been conducted to evaluate dichotomous keys in practice of key use (Morse et al. 1996) or to improve the key based on the user-tracking method (Schmidt et al. 2010). According to Osborne (1963), in principle, a simple dichotomous key used by an accurate observer must always lead to correct identification provided that the specimen in hand does actually belong to one of the taxa covered by the key and is not missing any crucial characters. Sandvik (1976) came to the conclusion that keys in which all taxa are gathered on the last two levels (so the number of steps of their identification is relatively equal) have the maximum probability of right determination. So our proposed index (E_Dicho_) can both evaluate the speed and the quality of the determination of a dichotomous key, provided that all else (e.g. choice of characters) being equal.

The E_Dicho_ index in its nature is an evenness index, therefore it has all the properties of a normal evenness index and is constrained between 0 and 1. The higher the variation in the number of steps we have to pass to come to the determination of the taxa, the lower is the E_Dicho_ index, and the asymptotic lowest value is 0. The highest value of 1 can be achieved in case of all the taxa having the same number of identification steps (Fig. 1.V). As we can see in the Figure 1, the two versions of the dichotomous key (1.I and 1.V) have the same number of taxa (8) and the same number of paired statements (7), but E_Dicho_ of the version ‘1.I’ is smaller than that of the version ‘1.V’, because the variation in the length of path of identification steps in the version ‘1.I’ is higher. Thus, the higher the E_Dicho_ index is, the “better” is the dichotomous key in the aspect of identification speed and in the aspect of right determination.

### An example of calculation of E_Dicho_ - the index for dichotomous keys

Let us consider five dichotomous keys as shown in the Figure 1.

Here, the number of taxa (S) equals 8. The number of steps or paired statements (Thesis + Antithesis) for identification of each taxon, the total number of steps for identification of all the taxa, and the proportion of steps to identify each taxon are the data for calculation of H’_Dicho_ of the dichotomous key and are presented in Table 1 for the five versions of the dichotomous key.

The calculation of H’_Dicho_ and E_dicho_ of five versions of the dichotomous key is presented in Table 2.

**Table 1.** The data for calculation of H’_Dicho_ for the keys in Figure 1.

Key version	The number of steps for identification of each taxon	The total number of steps for identification of all the taxa	The proportion of steps to identify each taxon
1.I	1,2,3,4,5,6,7,7	35	1/35,2/35,3/35,4/35,5/35,6/35,7/35,7/35
1.II	1,2,3,4,6,6,6,6	34	1/34,2/34,3/34,4/34,6/34,6/34,6/34,6/34
1.III	1,2,4,4,5,5,5,5	31	1/31,2/31,4/31,4/31,5/31,5/31,5/31,5/31
1.IV	2,2,3,3,4,4,4,4	26	2/26,2/26,3/26,3/26,4/26,4/26,4/26,4/26
1.V	3,3,3,3,3,3,3,3	24	3/24,3/24,3/24,3/24,3/24,3/24,3/24,3/24

**Table 2.** Calculation of H’_Dicho_ and E_dicho_.

Key version	H’_Dicho_	E_Dicho_= H’_Dicho_/ln(8)
1.I	-{(1/35).ln(1/35)+(2/35).ln(2/35)+(3/35).ln(3/35)+(4/35).ln(4/35)+ (5/35).ln(5/35)+(6/35).ln(6/35)+(7/35).ln(7/35)+(7/35).ln(7/35)}	0.937
1.II	-{(1/34).ln(1/34)+(2/34).ln(2/34)+(3/34).ln(3/34)+(4/34).ln(4/34)+ (6/34).ln(6/34)+(6/34).ln(6/34)+(6/34).ln(6/34)+(6/34).ln(6/34)}	0.943
1.III	-{(1/31).ln(1/31)+(2/31).ln(2/31)+(4/31).ln(4/31)+(4/31).ln(4/31)+ (5/31).ln(5/31)+(5/31).ln(5/31)+(5/31).ln(5/31)+(5/31).ln(5/31)}	0.959
1.IV	-{(2/26).ln(2/26)+(2/26).ln(2/26)+(3/26).ln(3/26)+(3/26).ln(3/26)+ (4/26).ln(4/26)+(4/26).ln(4/26)+(4/26).ln(4/26)+(4/26).ln(4/26)}	0.983
1.V	-{(3/24).ln(3/24)+(3/24).ln(3/24)+(3/24).ln(3/24)+(3/24).ln(3/24)+ (3/24).ln(3/24)+(3/24).ln(3/24)+(3/24).ln(3/24)+(3/24).ln(3/24)}	1.000

## Conclusions

By using computer software it is possible to create many dichotomous keys for a group of taxa with the same set of pairs of dichotomous characters. It would be desirable to have a sound basis for choosing one or another key version. The E_Dicho_ index developed here is suitable for a quantitative evaluation of dichotomous keys. It can serve well as the mathematical basis for the task of choosing the dichotomous key with the best performance. Because the index is based on the evenness, it can be used to compare the identification keys of different sizes.
